# Assessment of photoacoustic tomography contrast for breast tissue imaging using 3D correlative virtual histology

**DOI:** 10.1038/s41598-022-06501-3

**Published:** 2022-02-15

**Authors:** Gurneet S. Sangha, Bihe Hu, Guang Li, Sharon E. Fox, Andrew B. Sholl, J. Quincy Brown, Craig J. Goergen

**Affiliations:** 1 Fischell Department of Bioengineering, University of Maryland, 8278 Paint Branch Dr, College Park, MD 20742 USA; 2 Weldon School of Biomedical Engineering, Purdue University, 206 S. Martin Jischke Dr., West Lafayette, IN 47907 USA; 3 Department of Biomedical Engineering, Tulane University, 547 Lindy Boggs Center, New Orleans, LA 70118 USA; 4 Department of Pathology, LSU Health Sciences Center, New Orleans, 433 Bolivar St, New Orleans, LA 70112 USA; 5 Pathology and Laboratory Medicine Service, Southeast Louisiana Veterans Healthcare System, 2400 Canal Street, New Orleans, LA 70112 USA; 6 Delta Pathology Group, Touro Infirmary, 1401 Foucher St, New Orleans, LA 70115 USA; 7 Purdue University Center for Cancer Research, Purdue University, 201 S. University St., West Lafayette, IN 47907 USA

**Keywords:** Breast cancer, Cancer imaging

## Abstract

Current breast tumor margin detection methods are destructive, time-consuming, and result in significant reoperative rates. Dual-modality photoacoustic tomography (PAT) and ultrasound has the potential to enhance breast margin characterization by providing clinically relevant compositional information with high sensitivity and tissue penetration. However, quantitative methods that rigorously compare volumetric PAT and ultrasound images with gold-standard histology are lacking, thus limiting clinical validation and translation. Here, we present a quantitative multimodality workflow that uses inverted Selective Plane Illumination Microscopy (iSPIM) to facilitate image co-registration between volumetric PAT-ultrasound datasets with histology in human invasive ductal carcinoma breast tissue samples. Our ultrasound-PAT system consisted of a tunable Nd:YAG laser coupled with a 40 MHz central frequency ultrasound transducer. A linear stepper motor was used to acquire volumetric PAT and ultrasound breast biopsy datasets using 1100 nm light to identify hemoglobin-rich regions and 1210 nm light to identify lipid-rich regions. Our iSPIM system used 488 nm and 647 nm laser excitation combined with Eosin and DRAQ5, a cell-permeant nucleic acid binding dye, to produce high-resolution volumetric datasets comparable to histology. Image thresholding was applied to PAT and iSPIM images to extract, quantify, and topologically visualize breast biopsy lipid, stroma, hemoglobin, and nuclei distribution. Our lipid-weighted PAT and iSPIM images suggest that low lipid regions strongly correlate with malignant breast tissue. Hemoglobin-weighted PAT images, however, correlated poorly with cancerous regions determined by histology and interpreted by a board-certified pathologist. Nuclei-weighted iSPIM images revealed similar cellular content in cancerous and non-cancerous tissues, suggesting malignant cell migration from the breast ducts to the surrounding tissues. We demonstrate the utility of our nondestructive, volumetric, region-based quantitative method for comprehensive validation of 3D tomographic imaging methods suitable for bedside tumor margin detection.

## Introduction

Approximately one in eight women will develop invasive breast cancer during their lifetime^1^, highlighting a clinical need to develop advanced imaging methods that improve breast cancer characterization and diagnosis. Generally, lumpectomy is the gold standard for breast cancer removal. However, this lengthy and expensive surgical procedure results in a reoperative rate of 10–70%, depending on the surgeon’s experience and tumor complexity^2–6^. The breast tissue excised during lumpectomy typically undergoes histological analysis to characterize the tissue margin. Cancerous tissue with less than 2 mm of healthy tissue surrounding the edge, referred to as a close margin, or cancerous tissue touching the edge of the resection specimen, referred to as a positive margin, commonly results in the patient undergoing another lumpectomy procedure to remove all tumor tissue. Cytological examination and frozen sectioning are the current gold standards, but both methods are time-consuming and have suboptimal accuracy^6,7^. Therefore, the clinical problem is two-fold as positive margins are detected after the procedure leading to relatively high reoperative rates. Additionally, intraoperative histological analysis is a destructive process that limits post-operative histological evaluation. There is a clear clinical need to develop nondestructive intraoperative methods for rapid tumor margin detection to reduce reoperative rates and improve patient outcomes.

In the past decade, numerous reports have described prospective methods to improve intraoperative breast tissue characterization^6,8^. The problem is that a single technique cannot rapidly distinguish tumor margins with high sensitivity and deep tissue penetration. Radio frequency spectroscopy and ultrasound imaging can provide real-time imaging to reduce intraoperative time, but neither provide compositional or molecular information that can enhance cancer tissue detection^9–13^. Near-infrared fluorescence imaging has shown potential in providing in vivo information during breast cancer removal^14–16^, but requires exogenous contrast agents that still must be optimized for targeting efficiency, toxicity, and specificity, leading to regulatory approval challenges. Likewise, diffuse optical tomography has also been developed for in vivo breast cancer characterization but lacks the necessary resolution required to assess tumor margins^17,18^. Other optical techniques, such as diffuse reflectance imaging, optical coherence tomography, and Raman spectroscopy can provide high-resolution images to better characterize breast tumor margins, but these methods generally have long image acquisition times and suboptimal penetration depths^19–25^.

Photoacoustic tomography (PAT) has emerged as a promising label-free method to improve breast margin characterization by providing compositional information with superior penetration depth than conventional optical techniques^26–29^. The photoacoustic effect relies on interactions between pulsed laser light and tissue chromophores that generate acoustic waves acquired to reconstruct an image. The wavelength of pulsed laser light is tuned to visualize tissue composition, such as hemoglobin and lipid distribution at 1100 nm and 1210 nm light^26,30^. When combined with conventional ultrasound imaging, the user can visualize both breast tissue structure and composition. It is hypothesized that PAT can differentiate non-cancerous lipid-rich and abnormally vascularized hemoglobin-rich cancerous tissue^8,31–33^. Multiple reports support that dual-modality photoacoustic and ultrasound imaging may improve intraoperative breast cancer detection^26–28^. More recently, a conflicting study suggested that ex vivo PAT imaging of hemoglobin distribution correlates poorly with ultrasound-determined tumor location^34^. Comparing gold-standard histology, clinical imaging, and photoacoustic data is inherently challenging due to image co-registration issues. Thus, these conflicting PAT reports may be partially due to a lack of robust co-registration methods to accurately compare histology or gold-standard clinical imaging results with photoacoustic imaging datasets at the same location.

Inverted selective plane illumination microscopy (iSPIM) is an advanced optical imaging technique and variant of light-sheet microscopy that can help address image co-registration challenges for the validation of volumetric label-free imaging modalities. iSPIM uses an excitation light sheet to illuminate a plane of tissue, inducing a fluorescence emission collected using a detection objective. This approach allows users to acquire high-resolution volumetric datasets with imaging rates 30 × faster compared to conventional microscopy techniques^35^. Although iSPIM has limited depth penetration for intraoperative use, combined with tissue clearing techniques, it can provide large volume datasets with histological contrast and resolution that can be co-registered to lower resolution label-free techniques^36–42^. These high-resolution volumetric datasets can provide structural information regarding ducts and blood vessels, lipid distribution, and cellular density. Taken together, iSPIM can help coordinate PAT image co-registration with gold-standard histology, and additionally provide independent volumetric high-resolution histological confirmation of sources of contrast present in 3D label-free PAT images.

**Figure 1:**
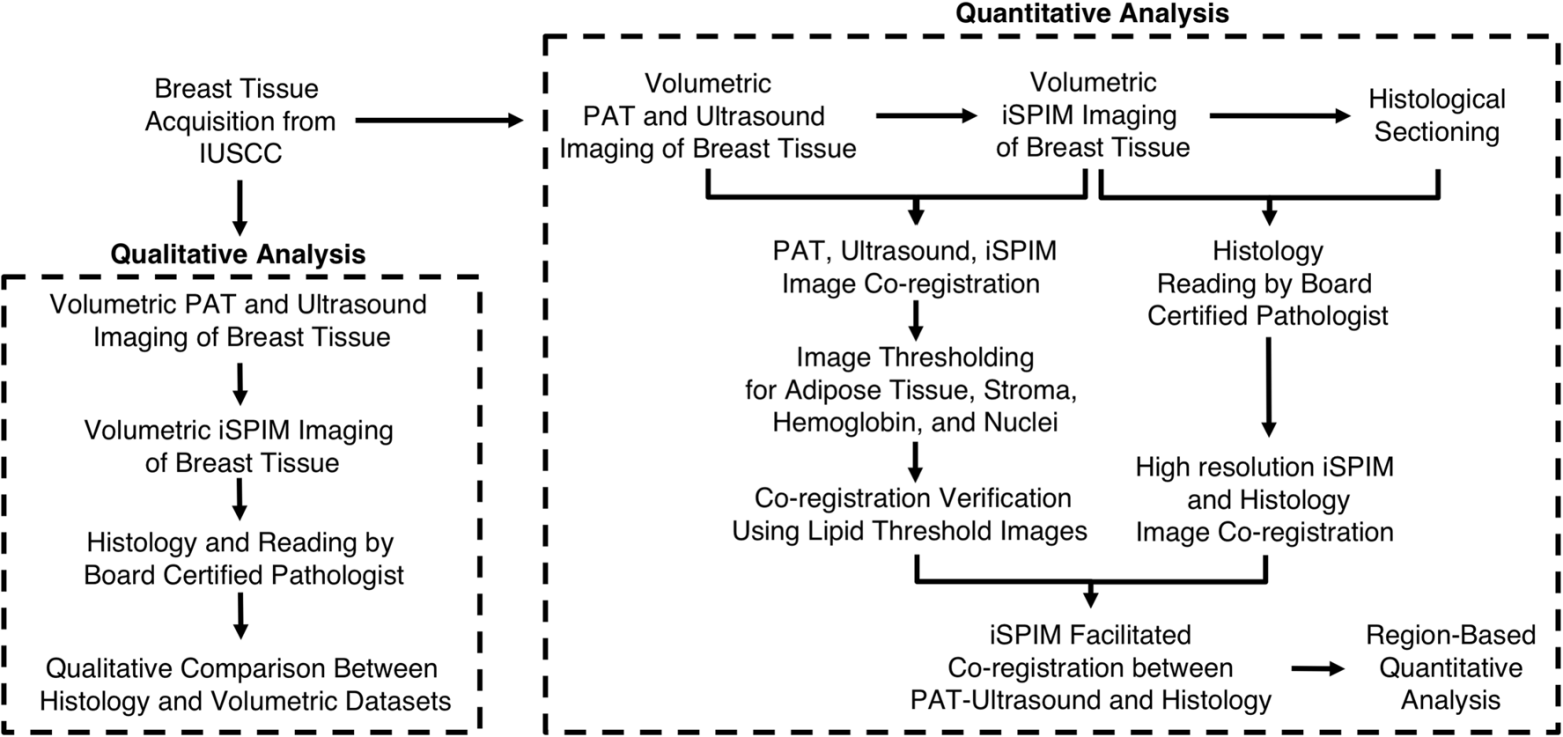
Overview of multimodality workflow used to characterize human invasive ductal carcinoma breast tissues. Qualitative breast tissue analysis was first performed by co-registering PAT, ultrasound, and iSPIM images with histology using overall morphology, internal structures, and composition. Quantitative breast tissue characterization was performed by verifying PAT-ultrasound and iSPIM co-registration using lipid-weighted threshold images, as well as co-registering high-resolution iSPIM images with histology. We then used iSPIM to facilitate co-registration between PAT-ultrasound and histology to assess how tissue composition changes in cancerous and non-cancerous regions.

Here, we present a novel multimodality imaging workflow to quantify the utility of PAT for breast biopsy characterization (Fig. 1). To do this, we developed a method using high-resolution iSPIM imaging to facilitate image co-registration between PAT-ultrasound datasets and gold-standard histology. We chose rapid, high-resolution PAT and ultrasound as a proposed method for depth-resolved tumor margin characterization due to their ease of integration that provides both tissue structure and label-free composition. Additionally, we use co-registered iSPIM volumes for independent 3D histological validation due to its rapid, large field of view imaging capabilities while being compatible with conventional sample mounting. We take advantage of our nondestructive volumetric imaging datasets and also quantify tissue composition and distribution in multiple regions throughout the breast tissue sample. Finally, we describe the current challenges regarding clinical multimodality breast tissue imaging and potential solutions using emerging engineering approaches.

## Methods

### Tissue acquisitions and preparation

Six breast cancer tissues from distinct patients were acquired from the Indiana University Simon Cancer Center (IUSCC) Tissue Bank. All six samples were imaged and three heterogenous biopsies were chosen for quantitative analysis. Samples were excised from female patients who underwent lumpectomy procedures and were subsequently diagnosed with infiltrating/invasive ductal carcinoma. Tissues were initially frozen followed by fixation in 4% paraformaldehyde for 24 h. These samples were then submerged in 0.1% paraformaldehyde and stored in a 4 °C refrigerator until imaged.

### Photoacoustic tomography

A custom designed PAT system was used to acquire 3D compositional information from the breast cancer biopsies (Supplementary Fig. S1). This system consisted of a high-resolution small animal ultrasound system (Vevo2100, FUJIFILM VisualSonics) with a 128 element, 40 MHz central-frequency probe coupled with a Nd:YAG optical parametric oscillator laser (Surelite EX, Continuum). Nd:YAG laser delivered a 10 Hz, 5 ns pulse ranging from 670 to 2300 nm to the tissue through a fiber optic bundle. The fiber optic bundle had an opening diameter of 1.0 cm and rectangular terminals of 12 × 2 mm^2^. These rectangular terminals were attached to the sides of the ultrasound transducer to couple light delivery and ultrasound-PAT signal acquisition. Synchronization of pulse delivery and signal acquisition was performed using a function generator (33220A, Agilent) that delivered a 10 Hz, 10 µs transistor-transistor logic signal to the laser and ultrasound system. PAT and ultrasound co-registration was achieved using a delay generator (DG535, Stanford Research System). For this study, we used 1100 nm light to produce hemoglobin-specific contrast, 1210 nm light to produce lipid-specific contrast, and 1400 nm light as an off-resonance control.

To minimize tissue movement during 3D scans, the breast cancer biopsies were fixed in place using 1% low-melt agarose in a silicon petri dish. Deionized water was used as an acoustic coupling media to eliminate bubble artifacts that are commonly observed with ultrasound gel. A MATLAB algorithm was then used to control a 3D step-wise motor to translate the PAT probe across the breast cancer biopsy in the y-direction and acquire both PAT and ultrasound images. The 3D motor was moved 0.193 mm across the sample and then paused to acquire 10 PAT images. This process was repeated for the anterior and posterior sides of the breast biopsies. Once the entire sample was imaged, the data was exported and processed using median averaging of 10 images for every location.

### Inverted selective plane illumination microscopy

The ASI iSPIM system has been described in our previous publications and is also illustrated in Supplementary Fig. S2^41^. This system was equipped with 488 nm and 647 nm laser sources (Omicron), fiber-coupled laser scanner (ASI) to generate a scanned light sheet, multi-immersion cleared tissue objectives (CTO, ASI/Special Optics) and CMOS cameras (ORCA-Flash 4.0, Hamamatsu). Two immersion objectives were aligned perpendicular to each other, with each having a 45° angle to the horizontal plane^41^. Both objectives switched roles between illumination and detection, making isotropic resolution possible by deconvolving dual view stacks^40^. However, in this experiment a single view was utilized. The field of view was 0.8 mm by 0.8 mm when the refractive index of immersion solution was 1.46. When large samples were imaged, 3D imaging could be realized by using a motorized stage with sub-micron repeatability to reconstruct samples in three dimensions.

After ultrasound-PAT imaging, the breast tissue samples underwent X-CLARITY polymerization and electrophoresis accelerated clearing (XCLARITY, Logos Biosystems) for one week. All cleared samples were stained in 50 µM DRAQ5 solution diluted in phosphate buffered saline overnight (Biostatus, Ltd), and then in 2 mg/mL Eosin Y solution diluted with 80% ethanol for 30 min (Sigma). After the staining procedure, samples were rinsed three times with deionized water for one minute, X-CLARITY mounting solution for 10 min, and fresh X-CLARITY mounting solution for one additional hour. This process helps match the sample’s refractive index to the mounting solution. Samples were fastened on the bottom of the imaging chamber with silicone gel (Dowsil) and then imaged with the iSPIM system. During imaging, the step size between two light sheets was set as 2 μm, overlap between two strips was 15%, and the layer step size was 450 μm. A 488 nm laser source was used for illumination of the Eosin channel and a 647 nm laser source was used for the DRAQ5 channel. Since the iSPIM system is able to image the breast samples cleared by X-CLARITY within a depth of 2 mm, e imaged both the anterior and posterior sides of the breast tissue sample. After imaging, the image stacks of the light sheet strips were reconstructed in a customized MATLAB program^43^, then stitched with the FIJI stitching plugin^44^, converted to pseudo-color images similar to hematoxylin and eosin (H&E) stained slide^45^, and visualized in Amira^40,46^.

### Histology

After completion of ultrasound, PAT and iSPIM imaging, the breast cancer biopsies were sent for standard histological sectioning. Tissues were paraffin embedded and cut with a slice thickness of 4 μm for histological sections. We used a step size of 200 μm for qualitative comparison and 30 μm for quantitative analysis. The slides were stained using H&E and then underwent blinded review by a board-certified pathologist to identify cancerous and non-cancerous regions.

### Image processing for quantitative analysis

All volumetric datasets underwent image thresholding to assess the percent and spatial composition of the breast biopsies. We first matched H&E histology sections with ultrasound, PAT, and iSPIM datasets based on the overall tissue morphology, internal structures (e.g., ducts), and composition. We then created iSPIM and PAT analysis regions consisting of one imaging slice matched with histology, as well as two imaging slices adjacent to the histology-matched imaging slice. A total of five analysis regions were created for each biopsy, consisting of three images that occupied a biopsy length of 0.386 mm.

The PAT images were cropped to the imaging depth of the iSPIM images prior to post-processing. Both datasets initially underwent edge-aware local contrast enhancement to improve morphological contrast and identification of structural edges. We then applied image binarization using Otsu’s method to identify PAT-specific lipid and hemoglobin pixels^47^. The iSPIM datasets underwent conventional thresholding using the image histogram to identify a threshold point in each region. The threshold points were averaged between regions and applied to the dataset to discriminate between lipid and stromal tissue, as well as DRAQ5-specific pixels that represent nuclei. Finally, we created tissue composition maps by averaging the binarized biopsy images to topologically visualize tissue composition and calculate percent composition in each region.

We then assessed how breast tissue composition differs in cancerous and non-cancerous regions. To do this, we assigned non-cancerous (presumed healthy) and suspicious cancerous ROIs to our tissue composition maps. ROI size was defined as 1/3 of the 2 mm imaging depth, or 0.66 × 0.66 mm. We first placed 20 ROIs in suspicious cancerous breast tissue determined by a board certified pathologist. We then placed 30 ROIs in non-cancerous breast tissue with lipid, hemoglobin, or nuclei content. ROIs were scored 1–3 using the compositional positive pixels, calculated by summing the number of binarized images that expressed lipid, hemoglobin, or nuclei contrast for a given pixel. ROIs with no hemoglobin or lipid contrast were scored as 1, composition positive pixels between one or two were scored as 2, and composition positive pixels greater than or equal to two composing at least 50% of ROI were scored 3. Nuclei ROI with no cellular content were scored as 1, composition positive pixels between one and two were scored 2, and composition positive pixels greater than or equal to two were scored 3.

### Statistical analysis

Linear regression statistics was used to evaluate the correlation between iSPIM and PAT-derived lipid composition, as well as the correlation between iSPIM nuclei and PAT hemoglobin composition. Shapiro–Wilk normality test was performed on all lipid, hemoglobin, and nuclei composition datasets. Datasets that failed normality test underwent log(y) or 1/y transformations. One way analysis of variance (ANOVA) with a Tukey post-hoc test was then performed to determine statistical significance in lipid, nuclei, and hemoglobin composition amongst the biopsies and analysis regions. Kruskal–Wallis test with Dunn’s multiple comparisons test was performed on ROI analysis data. The diagnostic ability of lipid, nuclei, and hemoglobin composition was quantified by calculating the area under the curve from receiver operating characteristics (ROC) curves. Statistical significance was considered at *p* < 0.05. Data are shown as mean ± standard deviation.

## Results

### Qualitative multimodality image comparison

**Figure 2:**
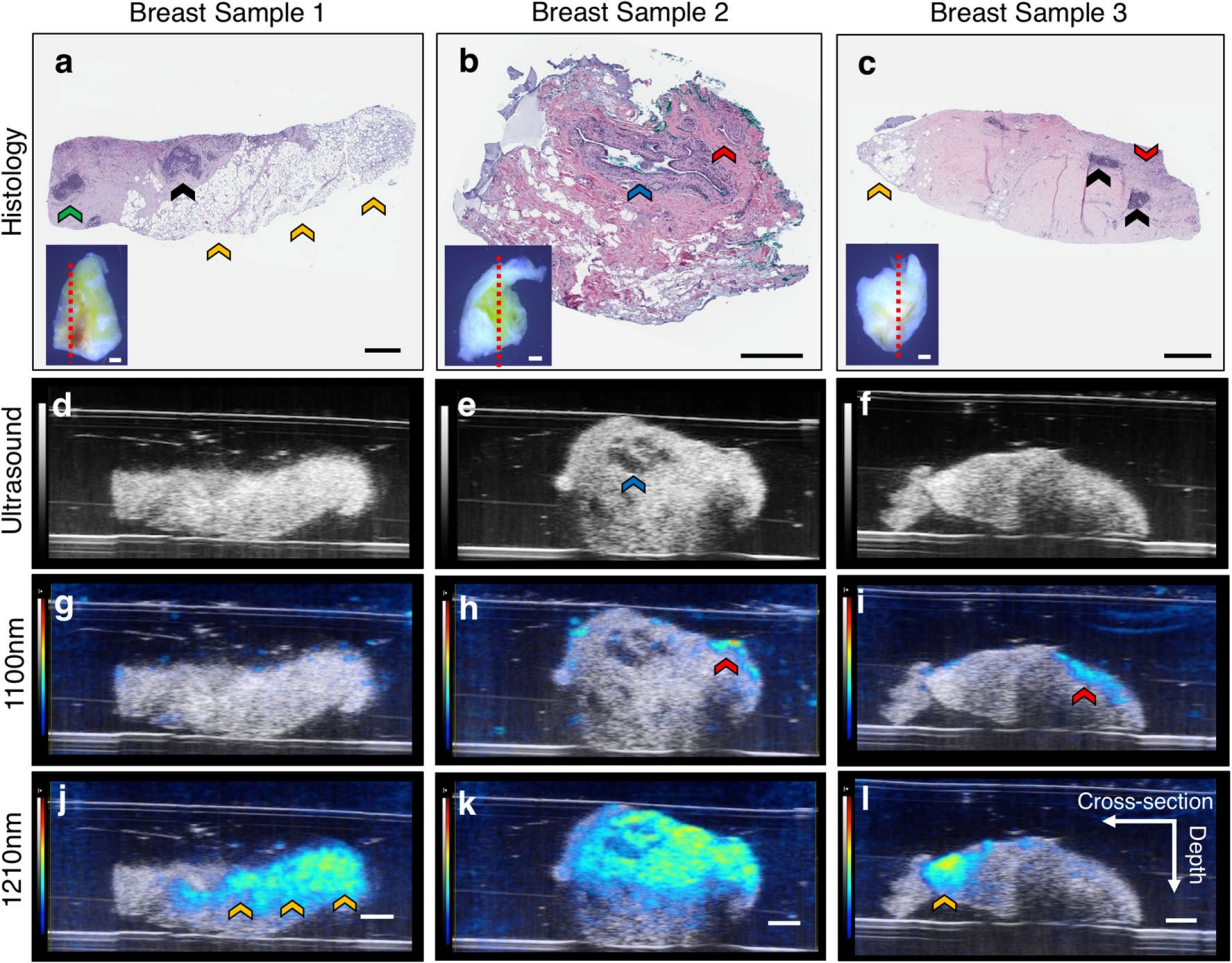
Representative histology (**a**–**c**), ultrasound (**d**–**f**), 1100 nm hemoglobin PAT (**g**–**i**), and 1210 nm lipid PAT (**j**–**l**) images of three breast tissue samples. Histological sections revealed structures including ducts (blue arrow), blood vessels (red arrow), chronic inflammation (green arrow), fatty tissue (orange arrow), and ductal cancer (black arrow). Ultrasound provided overall tissue morphology and visualization of specific structures such as ducts. Lipid-weighted PAT images allowed differentiation between adipose and stromal tissue, while hemoglobin-weighed PAT images provided contrast in regions with arteries and arterioles. Panel (**a**–**c**) insets depict breast sample images with the red dotted line indicating the image acquisition plane. All scale bars denote 1 mm.

Ultrasound and PAT images provided morphological and compositional information that was comparable to histology (Fig. 2a–c). Ultrasound images clearly resolved overall tissue morphology, including specific internal structures such as ducts (Fig. 2d–f). PAT images allowed for visualization of hemoglobin-rich regions via 1100 nm light (Fig. 2g–i) and lipid-rich adipose tissue via 1210 nm light (Fig. 2j–l). We see in sample 1 and 3 that lipid-rich tissues match well between histology and 1210 nm PAT images. Further, in sample 2 and 3 we see the ability of PAT to resolve large blood vessels (Fig. 2b,h), as well as smaller vessels that may feed the ductal carcinoma (Fig. 2c,i). Perfect co-registration in breast sample 2 was difficult between the lipid signal and traditional histology sections due to slight differences in the sectioning/imaging planes and varying step sizes. Adjacent histological sections to breast sample 2, however, reveal greater lipid-rich adipose tissue. Interestingly, breast sample 1 did not produce blood contrast surrounding the ductal carcinoma.

**Figure 3:**
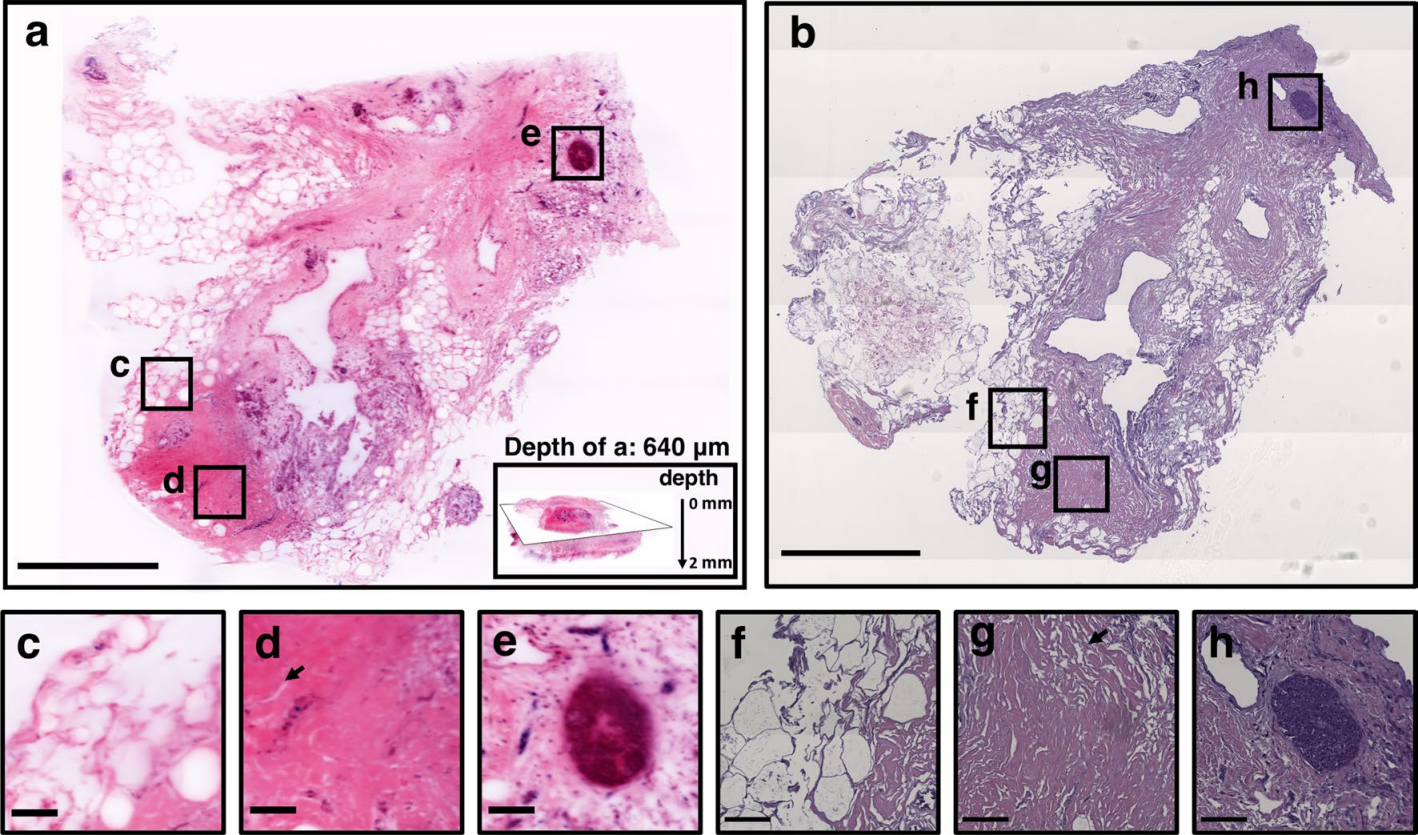
A transverse plane selected from the TVR of the iSPIM volumetric data (**a**) and a histological H&E section (**b**), both from the anterior side of the sample 2 biopsy. Overall, iSPIM images provided similar compositional and morphological information as H&E sections. We observed heterogeneous tissue composition with regions of adipose tissues (**c**,**f**), pseudoangiomatous stromal hyperplasia (**d**,**g**) and ductal carcinoma in situ (**e**,**h**). Scale bars of panel (**a**) and (**b**) denote 1 mm and scale bars of panel (**c**–**f**) denote 100 μm. The depth of this iSPIM volumetric image is 1.98 mm and sectioned iSPIM images (**a**,**c**,**d**,**e**) are shown at a depth of 640 μm; the total dimension of the image volume is 5.34 × 4.71 × 1.98 mm.

Comparison between iSPIM pseudo-color image and H&E-stained histological slides revealed similar compositional information (Fig. 3a,b). Texture-based volumetric rendering (TVR; Fig. 3a inset) was used to identify iSPIM images that match H&E histology. We were able to clearly identify nuclei structure and density, as well as spatially-resolve breast biopsy tissue composition. Indeed, iSPIM and H&E histology both revealed regions with adipose tissue (Fig. 3c,f), pseudoangiomatous stromal hyperplasia (Fig. 3d,g), and ductal carcinoma in situ (Fig. 3e,h)*.*

### Threshold validation and regional compositional analysis

**Figure 4:**
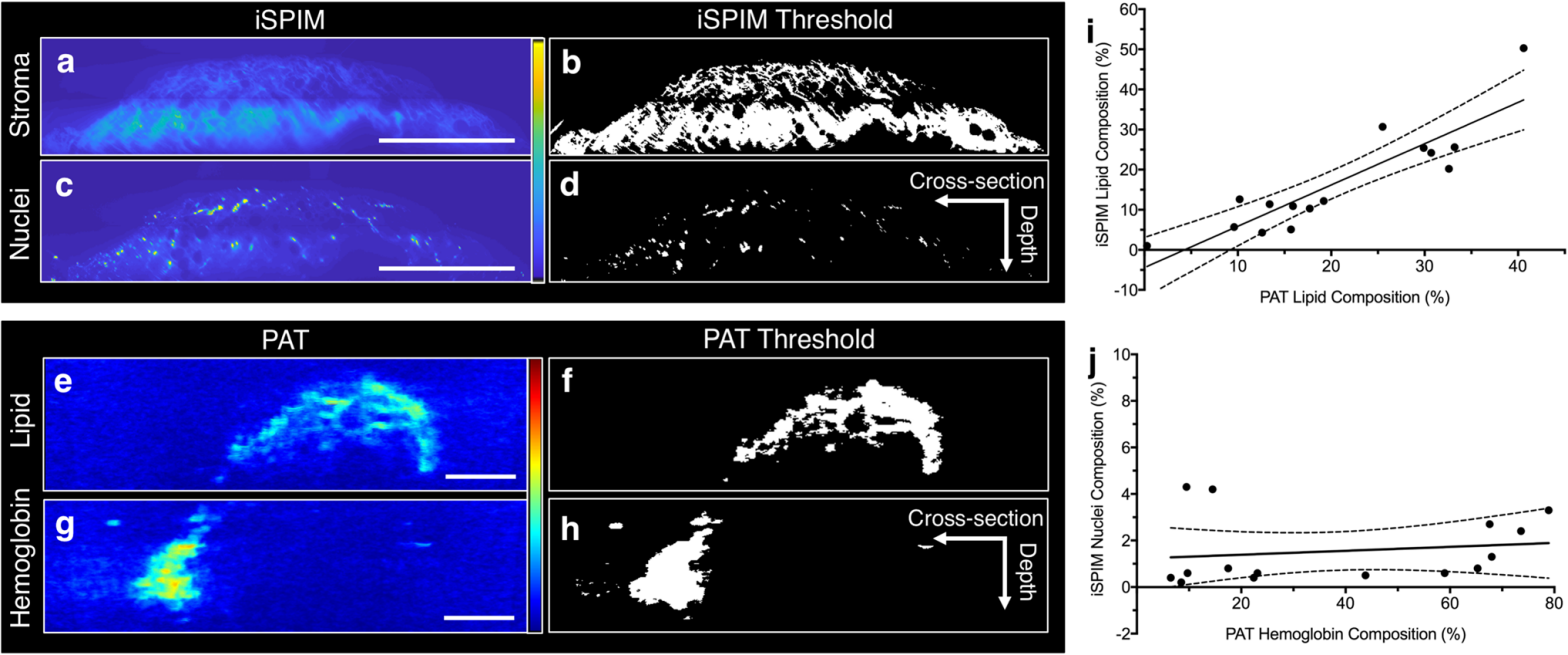
Volumetric iSPIM and PAT imaging datasets were acquired and underwent thresholding to extract compositional information. All datasets underwent edge-aware local contrast enhancement followed by conventional thresholding to distinguish iSPIM stroma (**a**,**b**) and nuclei (**c**,**d**) localization (n = 15). Otsu’s binarization was used to differentiate lipid-rich (**e**,**f**) and hemoglobin-rich (**g**,**h**) regions from the PAT data (n=15). Linear regression statistics between PAT and iSPIM lipid composition showed positive correlation (**i**, R^2^ = 0.78, *p* < 0.05). Additionally, linear regression statistics between PAT hemoglobin composition and iSPIM nuclei composition revealed no correlation (**j**, R^2^ = 0.03, *p* = 0.56). Scale bars denote 1 mm.

Image thresholding for breast biopsy composition adequately distinguished stroma (Fig. 4a,b), adipose tissue (Fig. 4e,f), nuclei (Fig. 4c,d), and hemoglobin-rich regions (Fig. 4g,h). We qualitatively identified comparable ultrasound, PAT, and iSPIM images using overall biopsy morphology and internal structures (e.g., ducts). A total of 15 regions were quantitatively analyzed, with each region comprising three image slices. This resulted in 45 total post-processed images. Co-registered images first underwent image thresholding to calculate the percent of the biopsy composed of stromal and lipid-rich adipose tissues. Linear regression revealed strong correlation in lipid composition between iSPIM and PAT data (Fig. 4i, R^2^ = 0.78, *p* < 0.05). Variation of tissue composition can be attributed to iSPIM stitching artifacts that were manually segmented and not included in the analysis. Interestingly, there was no correlation between hemoglobin and nuclei content in these biopsies (Fig. 4j, R^2^ = 0.03, *p* = 0.56), suggesting that nuclei dense regions do not necessarily coincide with hemoglobin-rich regions.

**Figure 5:**
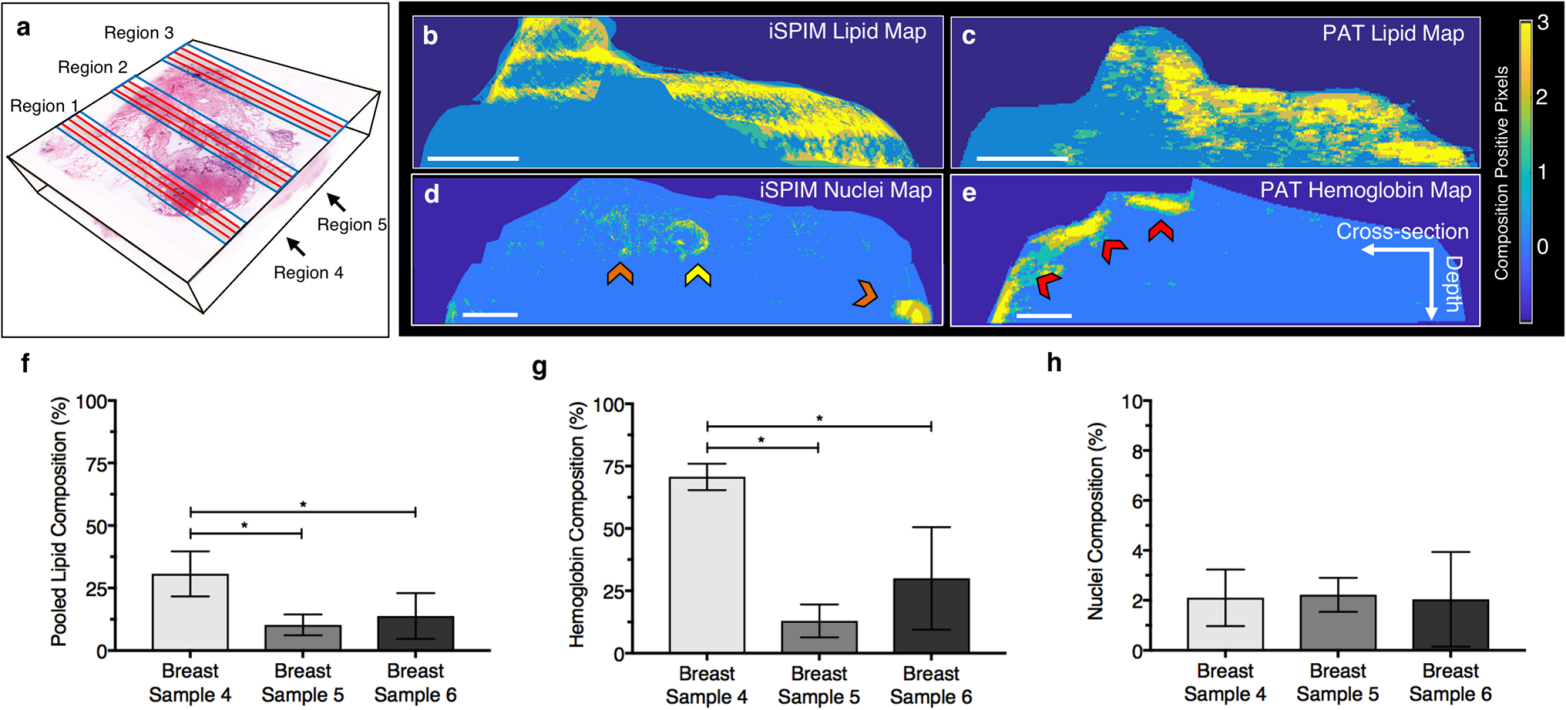
Quantitative analysis of breast tissue composition. A total of five regions (**a**, blue box) were identified from the anterior and posterior (**a**, black arrows) of three breast tissue samples. Each analysis region consisted of three iSPIM and PAT imaging slices (**a**, red lines). The three threshold imaging slices were averaged to obtain tissue composition maps of lipid (**b**,**c**), nuclei (**d**), and hemoglobin (**e**), as well as quantitative percent composition of lipid-rich tissue (**f**), hemoglobin (**g**), and nuclei (**h**). The tissue composition maps allow users to topologically visualize breast tissue composition within a sample region, as well as quantify tissue composition. Yellow arrow highlights nuclei located in a duct, orange arrows highlight nuclei in stromal tissue, and red arrow highlights hemoglobin-rich areas. Scale bar denotes 1 mm. * denotes *p* < 0.05 between groups. Error bars represent standard deviation.

We quantified breast tissue composition and averaged imaging slices within each region (Fig. 5a) to topologically visualize lipid-rich adipose and fibrotic stromal tissue distribution (Fig. 5b,c), as well as nuclei (Fig. 5d) and hemoglobin distribution (Fig. 5e). Pooled iSPIM and PAT lipid analysis results revealed average lipid composition of 30.6 ± 9.0% in breast sample 4, 10.3 ± 4.2% in breast sample 5, and 13.8 ± 9.1% in breast sample 6 (Fig. 5f). Hemoglobin contrast also varied greatly amongst different regions and samples, revealing 70.7 ± 5.8% in breast sample 4, 12.9 ± 6.6% in breast sample 5, and 30.0 ± 20.6% in breast sample 6 (Fig. 5g). Both lipid and hemoglobin composition were statistically greater in breast sample 4 than breast sample 5 and 6 (*p* < 0.05). Average nuclei content comprised 2.1 ± 1.3% of all three breast samples (Fig. 5h), but localization of cellular contrast varied greatly between regions and samples.

**Figure 6:**
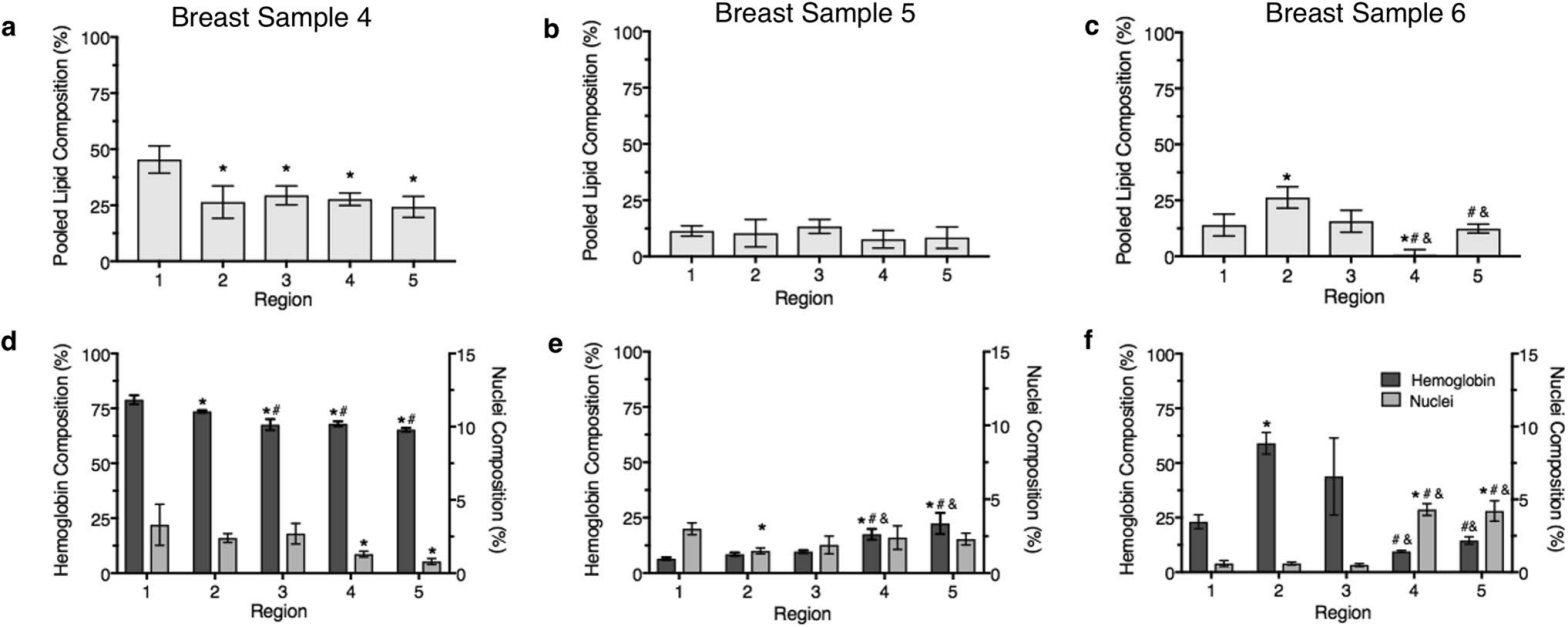
Quantitative regional analysis of breast tissue sample composition. Breast tissues samples were composed largely of stromal-rich tissues (**a**–**c**), while hemoglobin and nuclei composition widely varied depending on the tissue sample and region (**d**–**f**). Statistical significance determined at *p* < 0.05 (*statistical significance compared to region 1, ^#^statistical significance compared to region 2, and ^&^statistical significance compared to region 3). Error bars represent standard deviation.

Regional analysis confirmed comparable lipid and stromal composition between iSPIM and PAT images, with greater variation amongst nuclei and hemoglobin composition. Pooled lipid composition differed slightly between regions but varied amongst the three breast tissue samples. (Fig. 6a–c). We found that hemoglobin content was statistically greater in regions 1 (78.9 ± 2.0%) and 2 (73.6 ± 0.6%) than regions 3 (67.6 ± 2.5%), 4 (68.0 ± 1.2%), and 5 (65.3 ± 0.8%) from breast tissue sample 4; region 4 (17.5 ± 2.4%) and region 5 (22.4 ± 4.8%) than regions 1 (6.5 ± 0.6%), 2 (8.5 ± 0.8%), and 3 (9.7 ± 0.6%) in breast tissue sample 5; and regions 2 (59.0 ± 5.0%) and 3 (43.8 ± 17.7%) than region 4 (9.5 ± 0.50%) and 5 (14.5 ± 1.7%) in breast tissue sample 6 (Fig. 6d–f). Statistical analysis also revealed greater nuclei content in region 1 compared to regions 4–5 in breast tissue sample 4, and regions 4–5 compared to regions 1–3 in breast tissue sample 6 (Fig. 6d–f).

### Quantitative multimodality imaging and histology comparison

**Figure 7:**
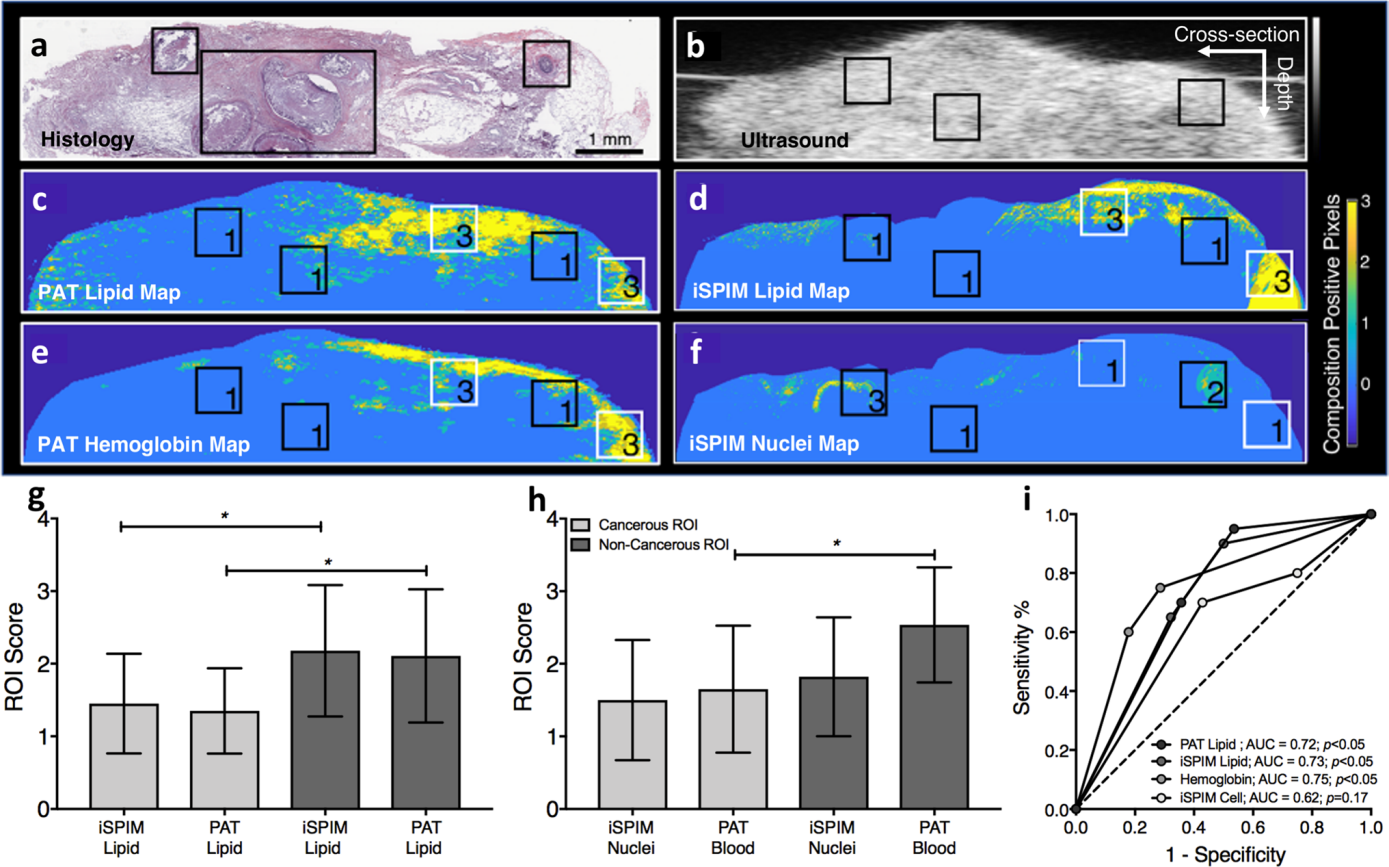
Comparison of lipid, hemoglobin, and nuclei composition in cancerous and non-cancerous breast tissues. A board certified pathologist first diagnosed suspicious tissue regions using H&E histology images (**a**). Ultrasound (**b**), PAT lipid (**c**), iSPIM lipid (**d**), PAT hemoglobin (**e**), and iSPIM nuclei (**f**) were used to assign cancerous (black box) and non-cancerous tissue (white box) ROIs. Each ROI was then assigned a score using predetermined rules. ROI analysis suggests that lipid rich regions correlate with non-cancerous breast tissue and stromal-rich regions correlate with cancerous tissues (**g**). Hemoglobin and nuclei content, however, did not positively correlate with regions with increased breast cancer prevalence (**h**). ROC area under the curve analysis suggests that lipid and hemoglobin contrast can help distinguish cancerous and non-cancerous region (**i**). *Denotes *p* < 0.05 between groups. Error bars represent standard deviation.

ROI analysis allowed us to compare breast sample lipid, hemoglobin, and nuclei composition in non-cancerous and cancerous regions (Fig. 7a–f). A board certified pathologist identified 20 tissue regions with suspicious cancerous features. Regions identified as non-cancerous had significantly higher lipid ROI scores compared to suspicious cancerous regions (Fig. 7g; *p* < 0.05). Specifically, suspicious cancerous lipid ROIs were scored 1.45 ± 0.69 using iSPIM lipid images and 1.35 ± 0.59 using PAT lipid images. Non-cancerous lipid ROIs were scored 2.18 ± 0.9 using iSPIM lipid images and 2.11 ± 0.92 using PAT lipid images. Non-cancerous and suspicious cancerous regions contained similar nuclei ROI scores of 1.82 ± 0.82 and 1.5 ± 0.83, respectively (Fig. 7h). Non-cancerous hemoglobin ROIs scored significantly higher at 2.54 ± 0.79 compared to suspicious cancerous regions at 1.65 ± 0.88 (Fig. 7h; *p* < 0.05). Finally, ROI analysis scores were then used to generate ROC curves (Fig. 7i), revealing area under the curve of 0.72 for PAT lipid (*p* < 0.05), 0.73 for iSPIM lipid (*p* < 0.05), 0.75 for hemoglobin (*p* < 0.05), and 0.62 for iSPIM cell (*p* > 0.05).

## Discussion

This study presents a multimodality imaging workflow to characterize breast tissue composition using a volumetric region-based quantitative method. The breast biopsies obtained for this study were classified as invasive ductal carcinoma, where cancer growing in the milk duct begins to infiltrate into surrounding stromal and adipose tissue. We first qualitatively assessed how PAT lipid and hemoglobin-contrast and iSPIM lipid and cellular distribution matched with gold-standard histology in three breast tissue biopsies. Dual-modality PAT and ultrasound allowed us to visualize the breast tissue sample morphology and differentiate stromal tissue from lipid-rich adipose tissue using 1210 nm light (Fig. 2j–l). PAT also provided hemoglobin contrast using 1100 nm light that correlated with regions with arterioles or large arteries (Fig. 2h,i). We chose a single near-infrared wavelength for lipid and hemoglobin imaging to minimize image acquisition time and maximize penetration depth. High-resolution iSPIM provided detailed, histology-like volumetric images that allowed visualization of nuclei-rich areas such as ducts and blood vessels and tissue composition, including adipose tissue (Fig. 3c,f), stroma (Fig. 3d,g), and ductal carcinoma in situ (Fig. 3e,h). We experienced challenges, however, when comparing volumetric datasets with traditional histology sections, due to variable image acquisition planes, histological artifacts, and substantial changes in breast tissue composition between 200 μm histology sections (Fig. 2b,k), supporting the need for iSPIM 3D histological imaging as a replacement for traditional histology sections in this context.

In three additional breast tissue biopsies, we created a systematic approach to compare multimodality imaging datasets and quantify lipid, stroma, nuclei, and hemoglobin distribution. PAT and iSPIM imaging slices obtained from the same volumes were first co-registered using overall tissue sample morphology, internal structures, and lipid distribution. High-resolution iSPIM images were also compared to gold-standard histology, allowing us to verify and use iSPIM as a 3D replacement for traditional histological validation. The three breast biopsies used for quantitative analysis were divided and analyzed into five subregions to topologically visualize and quantify tissue composition throughout the tissue. This approach allows the user to quickly visualize how lipid, stroma, hemoglobin, and nuclei are distributed throughout breast biopsy sub-regions compared to a representative histology image.

Image thresholding was performed to extract breast tissue composition in multiple tissue regions. Each thresholding method was tailored to a subset of PAT and iSPIM imaging slices and then applied to the entire breast tissue sample dataset. We found a positive correlation between iSPIM and PAT lipid composition (R^2^ = 0.78, *p* < 0.05), suggesting our thresholding method applied to label-free PAT imaging can be used to segment lipid-rich regions. Interestingly, we found no correlation between PAT hemoglobin composition and iSPIM nuclei composition (R^2^ = 0.03, *p* = 0.56), suggesting that iSPIM and PAT can provide unique compositional information to improve tissue characterization. In fact, the regional analysis showed that nuclei content comprises approximately 2.1 ± 1.3% of all three biopsies, while hemoglobin contrast varied from 12.9 ± 6.6% to 70.7 ± 5.8%. These results suggest iSPIM and PAT images can be co-registered using tissue morphology and composition to obtain lipid, stroma, hemoglobin, and nuclei distribution.

After validating our multimodality comparison method, we assessed tissue composition in cancerous and non-cancerous regions. We first assigned ultrasound, PAT, and iSPIM image ROIs in suspicious cancerous regions confirmed by a board-certified pathologist. We then placed ROIs in non-cancerous regions with lipid, hemoglobin, and nuclei contrast. ROIs were placed in the same location on the PAT, ultrasound, and iSPIM image and then scored using a predefined set of rules. Overall, our lipid-rich ROI scoring was not statistically different between iSPIM and PAT images, corroborating that PAT contrast at 1210 nm is lipid-specific and that we are comparing similar regions between volumetric datasets. Our ROI scoring and ROC area under the curve analysis suggest that lipid-rich regions are more likely to express non-cancerous phenotypes and regions with diminished lipid content are more likely to express cancerous phenotypes. This is consistent with histopathology reports that show that malignant cancerous cells migrate from the breast duct to the surrounding stroma, thus transforming ductal carcinoma in situ to invasive ductal carcinoma^48^. Cancer cell migration into adipose tissue may also decrease lipid content in the infiltrated region. Recent reports using PAT and broadband near-infrared mammography study found that reduced lipid concentration was an indicator of malignant breast tissue^33,34^. Therefore, lipid-weighted PAT images may enhance positive breast margin detection and allow clinicians to quantify cancer aggressiveness.

Pathologists routinely assess breast tumor grade using a modified Bloom-Richardson system to semi-quantitatively assess nuclear atypia^49^. Here, we visualized nuclei distribution throughout the breast tissue sample using image segmentation and then compared how nuclei distribution correlates with non-cancerous and cancerous breast tissue regions. Our ROI analysis suggested that nuclei distribution was similar in both non-cancerous and cancerous breast tissue regions. This data may indicate cancerous cells infiltration from the ducts into the surrounding non-cancerous tissues, but healthy control breast biopsies are needed to confirm this hypothesis. ROC area under the curve analysis also suggests limited diagnostic ability of iSPIM nuclei contrast alone. The diagnostic ability of nuclei weighted images may be improved by combining our method with automated segmentation techniques to quantify heterogeneous cancer nuclei characteristics such as enlarged and irregularly shaped nuclei throughout the breast biopsy^50^.

Interestingly, non-cancerous regions also showed greater PAT hemoglobin-weighted contrast compared to cancerous regions. This result contradicts the previously proposed hypothesis that regions with increased PAT hemoglobin contrast may indicate high-vascularized cancerous regions^31–33^. Excised breast biopsies lack dynamic blood flow causing blood vessels to collapse and altering blood distribution within the tissue sample. Additionally, hemoglobin distribution is likely altered through the tissue handling process^8,34^, which in this study includes washes after tissue resection, fixation, agarose embedding, and imaging with multiple modalities. Hemoglobin redox state also changes in excised tissues, causing oxygenated and deoxygenated hemoglobin to slowly convert into methemoglobin^51^. Changes in hemoglobin oxygenation and redox state will alter hemoglobin absorption spectra and subsequent PAT contrast^42^. Our results are also supported by Kosik et al*.*, who reported that 690-nm deoxyhemoglobin-weighted PAT images correlated poorly with ultrasound-determined cancer regions in freshly excised biopsies^34^. These factors highlight a need to establish breast biopsy preservation guidelines to preserve hemoglobin distribution and redox state after tissue resection.

The dual-modality PAT-ultrasound imaging used in this study can be further improved for intraoperative breast tumor analysis. We used high-resolution ultrasound to facilitate iSPIM and PAT co-registration; however, ultrasound elastography can be adapted to provide tissue stiffness measurements that may further improve diagnostic accuracy^52–54^. Bae et al*.* recently used shear-wave elastography to measure mean and maximum stiffness and elasticity ratio in 228 breast lymph nodes obtained from 55 patients^52^. Their results suggest that metastatic breast lymph nodes are significantly stiffer than non-metastatic breast tissue lymph nodes. The elasticity ratio was also higher in metastatic lymph nodes compared to non-metastatic lymph nodes. This pilot study highlights the potential of ex vivo shear wave elastography to enhance intraoperative breast cancer characterization.

PAT image quality is dependent on the light penetration, as photons are more likely to be scattered and absorbed when traveling deep inside tissue. Therefore, heterogeneous light distribution in tissue limits PAT image contrast quantification as superficial tissue absorbs more light and generates greater PAT signal than deep tissue. To our knowledge, PAT transducer frequency and illumination geometry have not been optimized for PAT signal generation in the first 2 mm of the breast biopsy. Previous PAT studies characterize cancerous breast tissue using 2, 12, and 21 MHz center frequency transducers^26,32,55^, while our study used a 40 MHz transducer. Similar to ultrasound, PAT imaging that relies on higher frequency acoustic waves acquisition provides superior resolution, but diminished penetration depth compared to lower frequency acoustic waves acquisition. Additionally, the photoacoustic signal is the product of the physical properties of the tissue known as the Grüneisen coefficient, tissue-specific optical absorption coefficient, and light fluence. The optical absorption coefficients and Grüneisen coefficients have has been characterized for many different issues, but there is still room to optimize light illumination geometry to maximize light fluence into the region of interest^56,57^. Therefore, transducer frequency and illumination geometry studies should be performed to obtain the greatest penetration depth and signal-to-noise ratio in the first 2 mm of the breast biopsy tissue. Optimized photoacoustic illumination geometries coupled with deep learning algorithms can enhance image contrast in low-fluence regions and sparse data^58,59^.

Light penetration also affects iSPIM penetration depth. More effective tissue clearing methods, including Ethyl Cinnamate clearing (ECi), clearing-enhanced 3D (Ce3D), and clear, unobstructed brain/body imaging cocktails (CUBIC), can help reduce light scattering and increase the signal-and-noise ratio in deep tissue regions. These methods have been previously used for human kidney, lung, and prostate tissue^21,60,61^. Enhanced image contrast from optically cleared tissues may also serve as controls to show that the wavelength used during PAT provides contrast from the correct tissue type^62^. Furthermore, the limited iSPIM field of view requires that multiple sub-volumes are stitched to reconstruct 3D datasets. iSPIM stitching introduces image artifacts that limit quantitative analysis. The stitching artifact could be diminished by using stitching algorithms or acquisition techniques such as electronic confocal slit detection or structured illumination, to enhance image contrast in deeper tissue regions^41^. Together, these methods enable more comprehensive supplements to help validate PAT for breast tumor margin detection.

Multimodality approaches inherently require additional manpower to acquire, post-process, and interpret volumetric datasets, which can quickly increase intraoperative margin assessment. Artificial intelligence has the potential to significantly improve multimodality intraoperative workflow by deciding which modalities are needed to assess breast margins, improving image quality, and automating complex volumetric image analysis. Li et al*.* calculated a theoretical PAT image acquisition rate of approximately 36 cm^2^/min using two wavelengths through a 10 Hz laser, with a 15 mm × 15 mm illumination area and a 200 μm translational step size^26^. On the other hand, iSPIM has a theoretical image acquisition rate of 0.008 cm^2^/min after sample staining and clearing. In the future, it may be possible to use PAT for initial breast margin assessment and then use iSPIM imaging to acquire high-resolution datasets in smaller rapidly-cleared regions where artificial intelligence-determined margin assessment is unclear. Moreover, manual thresholding and image segmentation of large volumetric datasets was labor-intensive in this study and exacerbated by imaging artifacts. Machine learning algorithms can improve image quality by improving image contrast, decreasing background noise, and identifying image artifacts^58,63,64^. Artificial intelligence is actively being developed to analyze mammography and pathological images^65,66^. Pacilè et al. recently performed a multireader, multicase retrospectively study using 14 radiologists and artificial intelligence to assess 240 digital two-dimensional breast cancer mammography images^65^. The authors of this study found that artificial intelligence improved breast cancer diagnostic performance without prolonging the radiologists’ workflow. Data obtained from ultrasound, PAT, and iSPIM can be input into deep learning algorithms to differentiate positive and negative breast tissue margins. However, this approach requires quantitative methods to determine where the cancer is located within the biopsy, as presented in this article.

## Conclusion

We developed a systematic method that uses iSPIM to rigorously compare volumetric ultrasound and PAT with gold standard histological sections and demonstrate the use of virtual histological imaging as an independent validation tool for label-free volumetric imaging techniques. Diagnostic features, such as lipid and stromal distribution and hemoglobin and nuclei localization, were extracted from multiple breast tissue regions and topologically visualized. Our results suggest that PAT and iSPIM lipid contrast can differentiate cancer-rich stromal tissue and non-cancerous lipid-rich adipose tissue. Nuclei data showed similar cellular content in cancerous and non-cancerous tissue, potentially indicating cancer infiltration from the milk duct to the surrounding tissue. Finally, hemoglobin-weighted PAT images suggest that non-cancerous regions contain more hemoglobin contrast than cancerous regions, conflicting with previously published reports. Together, a multimodality approach has the potential to enhance breast tumor margin characterization compared to a single imaging technique. Our systematic method comparing multiple modalities can also advance artificial intelligence methods aimed at differentiating positive and negative breast tumor margins.

## Supplementary Information

Supplementary Information.

## References

[1] DeSantis CE (2019). Breast cancer statistics, 2019. CA. Cancer J. Clin..

[2] McCahill LE (2012). Variability in reexcision following breast conservation surgery. JAMA.

[3] Jeevan, R. *et al.* Reoperation rates after breast conserving surgery for breast cancer among women in England: Retrospective study of hospital episode statistics. *BMJ* **345**, (2012). 10.1136/bmj.e4505 PMC3395735 22791786

[4] Wilke LG (2014). Repeat surgery after breast conservation for the treatment of stage 0 to II breast carcinoma: A report from the National Cancer Data Base, 2004–2010. JAMA Surg..

[5] Schulman AM (2017). Reexcision surgery for breast cancer: an analysis of the American Society of Breast Surgeons (ASBrS) Mastery SM database following the SSO-ASTRO “no ink on tumor” guidelines. Ann. Surg. Oncol..

[6] McEvoy MP, Landercasper J, Naik HR, Feldman S (2018). Update of the American society of breast surgeons toolbox to address the lumpectomy reoperation epidemic. Gland Surg..

[7] St John, E. R. *et al.* Diagnostic accuracy of intraoperative techniques for margin assessment in breast cancer surgery. *Ann. Surg.* **265**, 300–310 (2017). 10.1097/SLA.0000000000001897 27429028

[8] Balasundaram, G. *et al.* Biophotonic technologies for assessment of breast tumor surgical margins—A review. *J. Biophotonics* **14**, e202000280 (2021). 10.1002/jbio.202000280 32951321

[9] Schnabel F (2014). A randomized prospective study of lumpectomy margin assessment with use of MarginProbe in patients with nonpalpable breast malignancies. Ann. Surg. Oncol..

[10] Thill M (2013). MarginProbe®: intraoperative margin assessment during breast conserving surgery by using radiofrequency spectroscopy. Expert Rev. Med. Dev..

[11] Thill M, Dittmer C, Baumann K, Friedrichs K, Blohmer J-U (2014). MarginProbe®–Final results of the German post-market study in breast conserving surgery of ductal carcinoma in situ. The Breast.

[12] Krekel NMA (2013). Intraoperative ultrasound guidance for palpable breast cancer excision (COBALT trial): A multicentre, randomised controlled trial. Lancet Oncol..

[13] Rahman RL, Puckett Y, Habrawi Z, Crawford S (2020). A decade of intraoperative ultrasound guided breast conservation for margin negative resection–Radioactive, and magnetic, and Infrared Oh My…. Am. J. Surg..

[14] Liu J, Guo W, Tong M (2016). Intraoperative indocyanine green fluorescence guidance for excision of nonpalpable breast cancer. World J. Surg. Oncol..

[15] Zhang C (2019). Methylene blue–based near-infrared fluorescence imaging for breast cancer visualization in resected human tissues. Technol. Cancer Res. Treat..

[16] Tummers QRJG (2014). Real-time intraoperative detection of breast cancer using near-infrared fluorescence imaging and Methylene Blue. Eur. J. Surg. Oncol..

[17] Flexman ML (2013). Optical biomarkers for breast cancer derived from dynamic diffuse optical tomography. J. Biomed. Opt..

[18] Choe R (2009). Differentiation of benign and malignant breast tumors by in-vivo three-dimensional parallel-plate diffuse optical tomography. J. Biomed. Opt..

[19] Nichols, B. S. *et al.* A quantitative diffuse reflectance imaging (QDRI) system for comprehensive surveillance of the morphological landscape in breast tumor margins. *PLoS One* **10**, e0127525 (2015). 10.1371/journal.pone.0127525 PMC4468201 26076123

[20] Wang M (2016). Gigapixel surface imaging of radical prostatectomy specimens for comprehensive detection of cancer-positive surgical margins using structured illumination microscopy. Sci. Rep..

[21] Glaser AK (2017). Light-sheet microscopy for slide-free non-destructive pathology of large clinical specimens. Nat. Biomed. Eng..

[22] Fereidouni F (2017). Microscopy with ultraviolet surface excitation for rapid slide-free histology. Nat. Biomed. Eng..

[23] Zúñiga WC (2019). Raman spectroscopy for rapid evaluation of surgical margins during breast cancer lumpectomy. Sci. Rep..

[24] Ha R (2018). Optical coherence tomography: A novel imaging method for post-lumpectomy breast margin assessment—A multi-reader study. Acad. Radiol..

[25] De Boer LL (2018). Towards the use of diffuse reflectance spectroscopy for real-time in vivo detection of breast cancer during surgery. J. Transl. Med..

[26] Li R (2015). Assessing breast tumor margin by multispectral photoacoustic tomography. Biomed. Opt. Express.

[27] Li, R. *et al.* High‐speed intra‐operative assessment of breast tumour margins by multimodal ultrasound and photoacoustic tomography. *Med. Dev. Sens.* **1**, e10018 (2018). 10.1002/mds3.10018 PMC6703831 31435620

[28] Goh Y (2020). Ultrasound guided optoacoustic tomography in assessment of tumor margins for lumpectomies. Transl. Oncol..

[29] Wong, T. T. W. *et al.* Fast label-free multilayered histology-like imaging of human breast cancer by photoacoustic microscopy. *Sci. Adv.* **3**, e1602168 (2017). 10.1126/sciadv.1602168 PMC5435415 28560329

[30] Sangha GS, Phillips EH, Goergen CJ (2017). In vivo photoacoustic lipid imaging in mice using the second near-infrared window. Biomed. Opt. Express.

[31] Ermilov SA (2009). Laser optoacoustic imaging system for detection of breast cancer. J. Biomed. Opt..

[32] Manohar S (2007). Initial results of in vivo non-invasive cancer imaging in the human breast using near-infrared photoacoustics. Opt. Express.

[33] Anderson, P. G. *et al.* Broadband optical mammography: Chromophore concentration and hemoglobin saturation contrast in breast cancer. *PLoS One* **10**, e0117322 (2015). 10.1371/journal.pone.0117322 PMC4363570 25781469

[34] Kosik I (2019). Intraoperative photoacoustic screening of breast cancer: A new perspective on malignancy visualization and surgical guidance. J. Biomed. Opt..

[35] Wu Y (2011). Inverted selective plane illumination microscopy (iSPIM) enables coupled cell identity lineaging and neurodevelopmental imaging in Caenorhabditis elegans. Proc. Natl. Acad. Sci..

[36] Torres R (2016). Three-dimensional morphology by multiphoton microscopy with clearing in a model of cisplatin-induced CKD. J. Am. Soc. Nephrol..

[37] Torres R, Vesuna S, Levene MJ (2014). High-resolution, 2-and 3-dimensional imaging of uncut, unembedded tissue biopsy samples. Arch. Pathol. Lab. Med..

[38] Xie, W. *et al.* Diagnosing 12 prostate needle cores within an hour of biopsy via open-top light-sheet microscopy. *J. Biomed. Opt.* **25**, 126502 (2020). 10.1117/1.JBO.25.12.126502 PMC7744172 33325186

[39] Liu JTC (2021). Harnessing non-destructive 3D pathology. Nat. Biomed. Eng..

[40] Hu B, Li G, Brown JQ (2019). Enhanced resolution 3D digital cytology and pathology with dual-view inverted selective plane illumination microscopy. Biomed. Opt. Express.

[41] Hu B, Bolus D, Brown JQ (2017). Improved contrast in inverted selective plane illumination microscopy of thick tissues using confocal detection and structured illumination. Biomed. Opt. Express.

[42] Tanaka N (2017). Whole-tissue biopsy phenotyping of three-dimensional tumours reveals patterns of cancer heterogeneity. Nat. Biomed. Eng..

[43] Li, G., Hu, B. & Brown, J. Q. An approach of 3D reconstruction for images by Dual-view Inverted Selective Plane Illumination Microscopy (diSPIM). in *Novel Techniques in Microscopy* NW5C-5 (Optical Society of America, 2019).

[44] Preibisch S, Saalfeld S, Tomancak P (2009). Globally optimal stitching of tiled 3D microscopic image acquisitions. Bioinformatics.

[45] Elfer, K. N. *et al.* DRAQ5 and eosin (‘D&E’) as an analog to hematoxylin and eosin for rapid fluorescence histology of fresh tissues. *PLoS One* **11**, e0165530 (2016). 10.1371/journal.pone.0165530 PMC5082869 27788264

[46] Stalling D, Westerhoff M, Hege H-C (2005). Amira: A highly interactive system for visual data analysis. Vis. Handb..

[47] Otsu N (1979). A threshold selection method from gray-level histograms. IEEE Trans. Syst. Man. Cybern..

[48] Rakha EA (2018). Invasion in breast lesions: the role of the epithelial–stroma barrier. Histopathology.

[49] Veta M, Pluim JPW, Van Diest PJ, Viergever MA (2014). Breast cancer histopathology image analysis: A review. IEEE Trans. Biomed. Eng..

[50] Lagree A (2021). A review and comparison of breast tumor cell nuclei segmentation performances using deep convolutional neural networks. Sci. Rep..

[51] Zijlstra, W. G., Buursma, A. & Meeuwsen-Van der Roest, W. P. Absorption spectra of human fetal and adult oxyhemoglobin, de-oxyhemoglobin, carboxyhemoglobin, and methemoglobin. *Clin. Chem.* **37**, 1633–1638 (1991). 1716537

[52] Bae SJ (2018). Ex vivo shear-wave elastography of axillary lymph nodes to predict nodal metastasis in patients with primary breast cancer. J. Breast Cancer.

[53] Sigrist RMS, Liau J, El Kaffas A, Chammas MC, Willmann JK (2017). Ultrasound elastography: Review of techniques and clinical applications. Theranostics.

[54] Berg WA (2012). Shear-wave elastography improves the specificity of breast US: the BE1 multinational study of 939 masses. Radiology.

[55] Toi M (2017). Visualization of tumor-related blood vessels in human breast by photoacoustic imaging system with a hemispherical detector array. Sci. Rep..

[56] Sowers T, Yoon H, Emelianov S (2020). Investigation of light delivery geometries for photoacoustic applications using Monte Carlo simulations with multiple wavelengths, tissue types, and species characteristics. J. Biomed. Opt..

[57] Sangha GS, Hale NJ, Goergen CJ (2018). Adjustable photoacoustic tomography probe improves light delivery and image quality. Photoacoustics.

[58] Antholzer S, Haltmeier M, Schwab J (2019). Deep learning for photoacoustic tomography from sparse data. Inverse Probl. Sci. Eng..

[59] Hariri A, Alipour K, Mantri Y, Schulze JP, Jokerst JV (2020). Deep learning improves contrast in low-fluence photoacoustic imaging. Biomed. Opt. Express.

[60] Glaser AK (2019). Multi-immersion open-top light-sheet microscope for high-throughput imaging of cleared tissues. Nat. Commun..

[61] Nojima S (2017). CUBIC pathology: Three-dimensional imaging for pathological diagnosis. Sci. Rep..

[62] Li, X. *et al.* Ultraviolet photoacoustic microscopy with tissue clearing for high-contrast histological imaging. *Photoacoustics* **25**, 100313 (2022). 10.1016/j.pacs.2021.100313 PMC8581572 34804794

[63] Hauptmann A (2018). Model-based learning for accelerated, limited-view 3-d photoacoustic tomography. IEEE Trans. Med. Imaging.

[64] Allman D, Reiter A, Bell MAL (2018). Photoacoustic source detection and reflection artifact removal enabled by deep learning. IEEE Trans. Med. Imaging.

[65] Pacilè, S. *et al.* Improving breast cancer detection accuracy of mammography with the concurrent use of an artificial intelligence tool. *Radiol. Artif. Intell.* **2**, e190208 (2020). 10.1148/ryai.2020190208 PMC8082372 33937844

[66] Celik Y, Talo M, Yildirim O, Karabatak M, Acharya UR (2020). Automated invasive ductal carcinoma detection based using deep transfer learning with whole-slide images. Pattern Recognit. Lett..

