# Supplementary material for: Assessment of photoacoustic tomography contrast for breast tissue imaging using 3D correlative virtual histology
Source: Sci Rep. 2022 Feb 15;12:2532. doi: 10.1038/s41598-022-06501-3 (PMC8847353; doi:10.1038/s41598-022-06501-3)
Supplement: Supplementary file 1 — Supplementary Information. [file 41598_2022_6501_MOESM1_ESM.pdf]

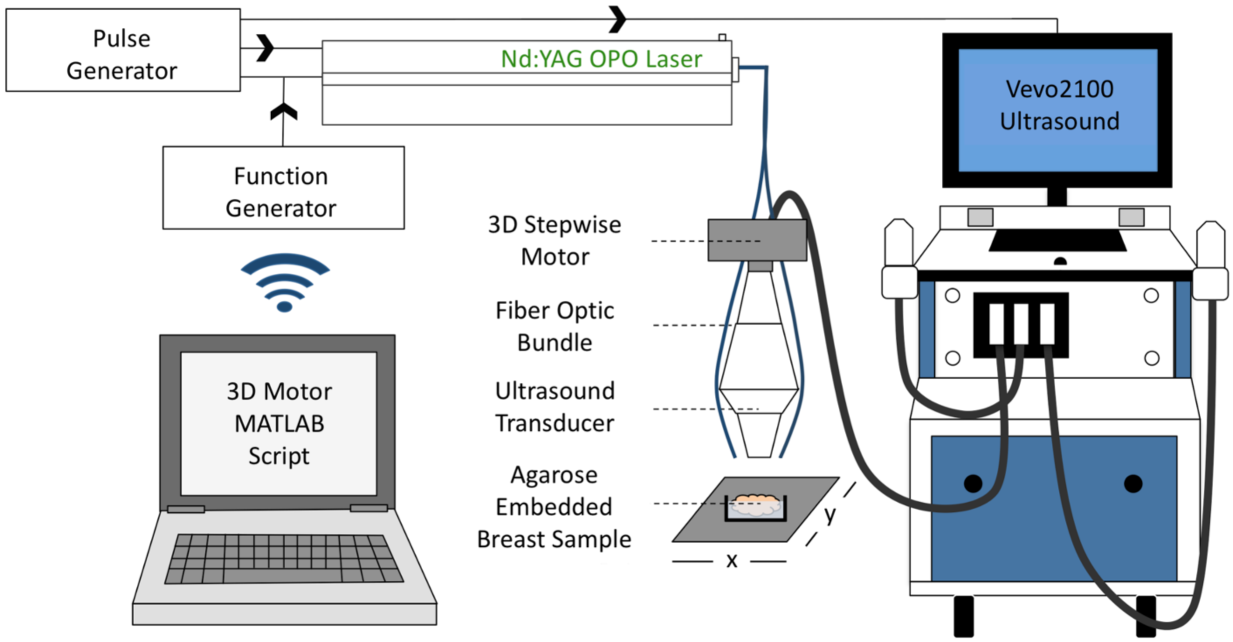

Supplementary Figure S1: Schematic of PAT-Ultrasound Setup. Pulse and function generator synchronized laser pulse excitation and PAT-Ultrasound signal acquisition. Custom MATLAB script controlled step-wise motor to acquire 3D PAT-Ultrasound volumes of human breast tissue sample.

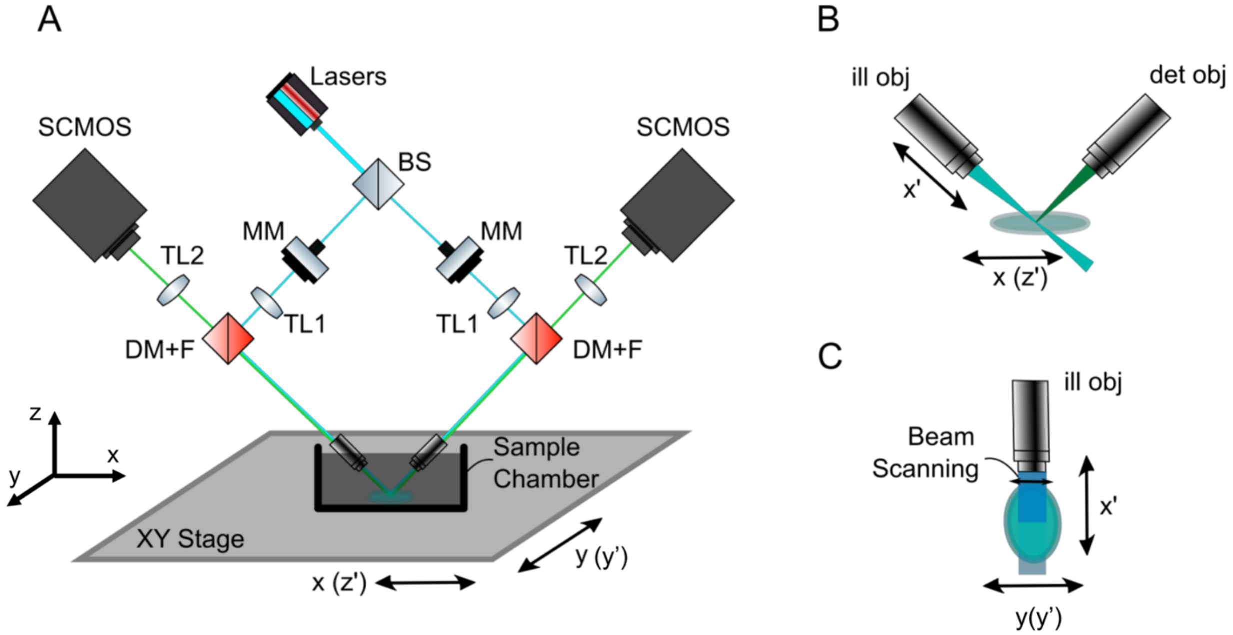

Supplementary Figure S2: iSPIM Schematic: A virtual light sheet generated by one of the micro-mirror scanners that scans in the y(y') direction rapidly illuminates the sample at an oblique 45° angle from one objective. The emission signal from the sample is then collected by the other objective perpendicular to the illumination objective and is imaged with the camera at the same side as the detection objective. Compared to the traditional xyz coordinates, a new set of coordinates of x'y'z' for iSPIM is used. x' axis is along the illumination direction, z' is the same as the traditional x axis which is the direction that the stage moves to create a 3D stack, and y' is the same with y. BS: beam splitter. MM: micro-mirror scanner. TL: tube lens. DM+F: dichroic mirror + emission filter.
